# Electrocardiographic and Echocardiographic Predictors of Atrial Fibrillation in Patients With Hypertrophic Cardiomyopathy

**DOI:** 10.3389/fcvm.2022.905128

**Published:** 2022-05-27

**Authors:** Leonard Mandeş, Monica Roşca, Daniela Ciupercă, Andreea Călin, Carmen C. Beladan, Roxana Enache, Andreea Cuculici, Cristian Băicuş, Ruxandra Jurcuţ, Carmen Ginghină, Bogdan A. Popescu

**Affiliations:** ^1^Cardiology Department, University of Medicine and Pharmacy “Carol Davila”- Euroecolab, Bucharest, Romania; ^2^Emergency Institute for Cardiovascular Diseases “Prof. Dr. C. C. Iliescu,” Bucharest, Romania

**Keywords:** hypertrophic cardiomyopathy, atrial fibrillation, echocardiography, electrocardiography, prognosis

## Abstract

**Background:**

Patients with hypertrophic cardiomyopathy (HCM) have an increased prevalence of atrial fibrillation (AF) compared to the general population, and left atrium (LA) remodeling is strongly correlated with the risk of AF. This prospective, monocentric study aimed to assess the role of LA electrocardiographic and echocardiographic (structural and functional) parameters in predicting the risk for incident AF in patients with HCM.

**Methods and Results:**

The study population consisted of 126 HCM patients in sinus rhythm (52.6 ± 16.2 years, 54 men), 118 of them without documented AF. During a median follow-up of 56 (7–124) months, 39 (30.9%) developed a new episode of AF. Multivariable analysis showed that LA booster pump function (assessed by ASr, HR = 4.24, CI = 1.84–9.75, and *p* = 0.038) and electrical dispersion (assessed by P wave dispersion – Pd, HR = 1.044, CI = 1.029–1.058, and *p* = 0.001), and not structural parameters (LA diameter, LA volume) were independent predictors of incident AF. Seventy-two patients had a LA diameter < 45 mm, and 16 of them (22.2%) had an AF episode during follow-up. In this subgroup, only Pd emerged as an independent predictor for incident AF (HR = 1.105, CI = 1.059–1.154, and *p* = 0.002), with good accuracy (AUC = 0.89).

**Conclusion:**

Left atrium booster pump function (ASr) and electrical dispersion (Pd) are related to the risk of incident AF in HCM patients. These parameters can provide further stratification of the risk for AF in this setting, including in patients considered at lower risk for AF based on the conventional assessment of LA size.

## Introduction

Patients with hypertrophic cardiomyopathy (HCM) have an increased prevalence of atrial fibrillation (AF) compared to the general population, and left atrium (LA) remodeling is strongly correlated with the risk of AF (1, 2).

Structural LA remodeling is related to the risk of AF in HCM patients, significant LA dilation being associated with an increased AF burden (3). Nevertheless, between 20 and 50% of HCM patients without significant LA dilation (LA diameter < 45 mm) will develop AF, suggesting that while LA diameter and LA volume are reproducible, easy to measure parameters, they may lack sensitivity to detect early atrial remodeling (3, 4).

Several studies have linked alterations in LA reservoir function (measured by total emptying fraction or by LA strain) and in LA booster pump function (quantified by volumetric parameters) with an increased risk for AF (4–6). The relationships between LA conduit function and AF or LA booster pump function assessed by speckle-tracking echocardiography (STE) and AF have not been studied. Compared to volumetric measurements, 2D STE-derived strain is more sensitive and less influenced by loading conditions (4–6).

There is also limited data regarding LA electrical remodeling assessed by simple and reproducible electrocardiographic (ECG) parameters such as P wave dispersion (Pd) or P wave maximal duration (Pmax) and the risk of AF in HCM patients. Two studies have shown a correlation between P wave parameters (Pd, Pmax) and paroxysmal AF, but these studies were retrospective, small, and excluded patients with a history of persistent AF (7, 8). Another study has evaluated the electromechanical delay of the LA (the duration between the onset of P wave on the ECG to the peak a’ wave of the lateral LA wall using tissue Doppler) in patients with HCM, showing that an increase in PA-TDI duration was associated with new-onset AF (9).

Atrial fibrillation is the most common arrhythmia seen in HCM patients, and its development is associated with a poor prognosis due to increased thromboembolic risk and worsening heart failure (HF), especially if it goes undetected (10, 11). Therefore, finding new, more sensitive AF predictors could help to identify HCM patients at risk, especially among those without significant LA dilation, that still have an increased AF burden compared with the general population.

Our study hypotheses were: (1) Patients with HCM and AF have significant electrical, functional, and structural LA remodeling compared to those without AF; and (2) in HCM patients, electrical and functional LA remodeling parameters are more accurate than structural parameters in predicting the risk of AF.

## Materials and Methods

### Study Population

We prospectively screened for eligibility consecutive patients referred to our echocardiography laboratory that met the current guidelines criteria for HCM: diastolic maximal wall thickness (MWT) of at least 15 mm in one or more LV myocardial segments evaluated by 2D and M mode echocardiography in the absence of a secondary cause for LV hypertrophy (3).

Patients with a history of ischemic heart disease (documented myocardial infarction or inducible ischemia with significant coronary artery stenosis in the absence of revascularization), previous cardiac surgery (by-pass, valvular prosthesis, valvuloplasty, and myomectomy), alcohol septal ablation, endocarditis, active neoplasia and with moderate/severe hepatic and renal failure were excluded. Only patients in sinus rhythm at the time of enrollment – both on ECG and at the initial Holter ECG monitoring – were included. ECG criteria for exclusion were permanent/persistent AF or atrial flutter, second or third-degree AV block, preexcitation, and paced atrial or ventricular rhythm during examination.

Patients with poor acoustic windows, unsuitable echocardiographic images for 2D STE analysis, moderate to severe valvular disease (except for mitral regurgitation related to functional and structural abnormalities characteristic for HCM), LV ejection fraction <50%, wall motion abnormalities and LV apical aneurysm were not included. For patients with significant arterial hypertension (systolic blood pressure > 160 mm Hg), additional criteria besides LV hypertrophy were required for inclusion: positive familial history or genetic testing for HCM (when available), specific ECG findings, severe LV hypertrophy (MWT > 20 mm), RV free wall hypertrophy, severe longitudinal dysfunction (e’ < 4 cm/s, s’ < 4 cm/s), or severe diastolic dysfunction. Patients with a familial history of HCM or positive genetic testing and mild LV hypertrophy (MWT = 13–14 mm) were not included.

In patients where we suspected a non-sarcomeric cause for HCM (e.g., amyloidosis, Fabry disease, and neuromuscular/mitochondrial diseases), additional testing was performed (specific biological testing, genetic testing, scintigraphy, or cardiac magnetic resonance). Patients where a secondary cause of HCM was identified or the clinical suspicion of a non-sarcomeric mutation remained high were excluded. Moreover, patients lost to follow-up (five patients) were excluded. The final study population consisted of 126 patients. Baseline characteristics and clinical data were collected at enrollment – age, sex, body mass index, history of AF, sudden death score, cardiovascular risk factors (smoking history, dyslipidemia, hypertension, and diabetes mellitus), relevant symptoms (angina, syncope, dyspnea, and palpitations), heart rate (HR), BP, and current medication (beta-blockers, calcium channel blockers, and antiarrhythmic medication). Hypertension was diagnosed and graded as recommended by current guidelines (12). Dyslipidemia was defined as a total cholesterol level > 200 mg/dl. Functional capacity was graded according to the New York Heart Association (NYHA) classification. All patients had standard laboratory testing (including renal function), and for 97 patients we assessed the brain natriuretic peptide (BNP) levels.

We prospectively followed the patients at 6 months intervals if they were free of symptoms until the first documented AF episode. This had to be confirmed by ECG, Holter monitoring, or device interrogation when available. Immediate evaluation was performed if clinical presentation suggested AF (sustained palpitations). Every patient in the study had at least one clinical, biological, and ECG/Holter ECG follow-up evaluation at 6 ± 1 months. In addition, patients with devices were also screened for AF at 6-months intervals through device interrogation. We also recorded HF worsening (defined as new hospitalization for HF or at least one NYHA class worsening) or BNP worsening (an increase of at least 50% from baseline).

All patients signed the informed consent for study participation and the study had the ethics committee’s approval.

### Electrocardiographic Study and Holter Monitoring

All patients had at least one standard, 25 mm/s, 12 lead ECG (simultaneously recorded, with 0.5–150 Hz filter, and AC filter 50 Hz) obtained in the supine position. ECG recordings were scanned and stored digitally. Measurements were performed digitally, on magnified recordings, using a digital caliper (EP caliper, version 2.6, EP Studios) that allows precise manual measurements.

#### P Wave Analysis

P wave duration (Pdur) was calculated in each lead as the time (expressed in ms) from the onset (defined as the junction between the isoelectric line and the first upward/downward departure from the baseline) to the end (the point of the return to baseline from the bottom/top of the trace) of the P wave. For the last digit in Pdur the values were rounded down to 0 or 5 ms (for values <2.5 and <7.5 ms, respectively) or rounded up to 5 or 10 ms (for values ≥2.5 and ≥7.5 ms, respectively). Pd was defined as the absolute difference between the maximal and the minimum Pdur (13, 14). The parameter Pamp was defined as the sum between the maximal absolute value of the two P wave components in V1 and the maximal amplitude of the P wave in DII (expressed in mV). Leads where the variations of the isoelectric line were larger than 50% of the maximal P wave amplitude were excluded from analysis.

#### Electrocardiographic Holter Monitoring

All patients had at least two (24 or 48 h) ECG Holter monitoring as screening for AF (on average 4.9 ± 2.2 ECG Holter recordings per patient) – at enrollment and at least one during follow-up, either at 6 or 12 months interval (as recommended by the attending physician), or earlier if clinically indicated (high suspicion of arrhythmia). AF was defined as the absence of P or f waves with an irregular heart rhythm of at least 30 s duration (for patients with supraventricular arrhythmias detected by implanted devices, only when the stored electrograms were suggestive for AF as decided by an expert; 15).

### Echocardiographic Study

A standard echocardiographic exam based on the European Association of Echocardiography recommendations was performed to all patients using a commercially available machine (Vivid 7, Vivid 9, or Vivid E95, General Electric Medical Systems, Horten, Norway) equipped with a M4S transducer (16). Conventional views were analyzed offline using a dedicated software (EchoPAC PC version 201; GE Medical Systems, Milwaukee, WI, United States) and 2D-STE analysis was performed as recommended (17). MWT was measured at end-diastole from LV short-axis views at basal, mid, and apical levels (18). Filling pressures were estimated from the ratio between peak early diastolic transmitral flow velocity E and e’ (calculated as the average of septal and lateral e’;^19^). Left ventricular diastolic dysfunction was assessed and graded according to current recommendations (20). Mitral regurgitation severity was graded semiquantitatively using Color Doppler echocardiography into trivial (grade 1), mild (grade 2), moderate (grade 3), and severe (grade 4) (21). Color Doppler and pulsed wave Doppler were used to explore for the presence of intraventricular gradient, while continuous wave Doppler was used to quantify obstruction severity (intraventricular gradient was considered significant if >30 mm Hg at rest; 3).

#### Left Atrium Analysis

The LA anteroposterior diameter (LAD) was determined from the 2D parasternal long-axis view, while LA maximal volume (LAV) was measured at end-systole from the apical four-chamber view, using the Simpson method (18). STE analysis of LA strain and strain-rate parameters was performed on an apical four-chamber view with the smallest sector width that included the LA walls. Patients with at least one segment with inadequate image quality were excluded from further analysis. Peak LA strain (ε) and strain rate, Sr (SSr – systolic, ESr – early diastolic, and ASr – late diastolic during atrial contraction) were measured as LA functional parameters, as recommended: SSr for reservoir function, ESr for conduit function, and ASr for booster pump function (22, 23; Supplementary Figure 1).

### Study Endpoint

The study end-point was defined as a new documented episode of AF (confirmed by a cardiologist from ECG, Holter monitoring, or device interrogation) regardless of AF history or clinical symptoms. In addition, HF worsening was recorded during follow-up visits.

### Statistical Analysis

Variables were reported as mean ± standard deviation. Variables between groups were compared using Student’s *t*-test, analysis of variance, Mann–Whitney *U* or *X*^2^ test when appropriate. The relationships between different parameters were assessed by correlation analysis. Cox proportional hazard regression analysis was performed to identify univariable associates for new-onset AF episodes. Standard receiver operator curves (ROC) and areas under curves (AUC) were calculated for every parameter independently associated with AF and used to establish cut-off values. All parameters with a two-sided *p*-value < 0.05 at univariable level that had an AUC > 0.6 were entered in the multivariable analysis, while adjusting for collinearity. Kaplan–Meier cumulative survival curves free of AF were constructed for all independent predictors for AF, stratified according to their cutoff values and compared by log-rank test. The likelihood ratio test was computed to explore the potential incremental value of adding additional parameters in a model to predict new-onset AF. All statistical analyses were performed using SPSS 26.0 software for Windows (SPSS, Inc., Chicago, IL, United States).

Measurement variability was assessed for Pd and Pamp (LM and AC), in a randomly selected group of 15 patients with HCM. Our lab’s measurement variability for LA strain parameters was previously reported (MR and AC; 17). For interobserver variability, measurements were carried out by a second operator on previously acquired images. For intraobserver variability, two sets of measurements were carried out by the same operator, 1 month apart.

## Results

### Study Participants

The final study population consisted of 126 patients. Of the patients included, 39 patients (30.9%) developed AF during follow up. Thirty-one patients developed their first documented episode of AF and eight patients with a history of paroxysmal AF in the past (documented by either ECG or Holter ECG monitoring before enrollment) developed a new episode of AF. Twenty-three patients had an implantable device (ICD/pacemaker). As expected, patients had increased wall thickness (median 21 mm), small LV indexed volumes, preserved EF, and diastolic dysfunction. Intraventricular obstruction (defined as resting gradient > 30 mm Hg) was present in 78 patients (61.9%), while functional mitral regurgitation (grade 2 or higher) was found in 54.7% of the patients. There were no significant differences between patients with and without AF regarding treatment with beta-blockers (94.8% vs 94.2%, *p* = 0.61), calcium channel blockers (9.7% vs 7.7%, *p* = 0.52), angiotensin-converting enzyme inhibitors (43.5% vs 28.7%, *p* = 0.075), or antiarrhythmic medication (amiodarone, 15.3% vs 10.3%, *p* = 0.44). There were significant but weak correlations between Pd and LAVi (*r* = 0.30, *p* < 0.001) and between Pd and functional LA parameters (*r* = 0.23, *p* = 0.012 for ASr and *r* = -0.29, *p* = 0.001 for systolic LA strain).

### Study Endpoint

During a median follow-up of 56 (7–124) months, 39 patients developed AF: 31 experienced new-onset AF, while 8 had a recurrence of AF (a new, stand-alone episode of AF in a patient with a history of paroxysmal/persistent AF). The AF episodes were diagnosed by ECG (*n* = 27), Holter monitoring (*n* = 9), or device interrogation (*n* = 3). Sixteen of these 39 patients (41%) had a LAD < 45 mm.

### Atrial Fibrillation Predictors in the Whole Study Population

Demographic, clinical, ECG, and echocardiographic characteristics of these patients are listed in Table 1. Intraobserver variability was 8.9 ± 9.2% for Pd, 23.2 ± 10.1% for Pamp, and 10 ± 9.6% for Pdur max. Interobserver variability for the same parameters was 16.9 ± 9.4%, 25.6 ± 11.3%, and 15.4 ± 8.1%, respectively.

**TABLE 1:** Demographic, clinical, ECG, and echocardiographic characteristics in the whole HCM population and in patients with and without atrial fibrillation during follow-up.

	Study population (*N* = 126)	HCM patients with AF (*N* = 39)	HCM patients without AF (*N* = 87)	*p*
**Demographic and clinical characteristics**				
**Age (years)**	**52.6 ± 16.2**	**58.6 ± 12.8**	**49.8 ± 16.9**	**0.002**
Men, *n* (%)	54 (42.8%)	15 (38.4%)	39 (44.8%)	0.221
BMI (kg/m^2^)	27.7 ± 4.8	28.1 ± 3.7	27.6 ± 5.3	0.583
SBP (mm Hg)	127 ± 20.4	127.8 ± 24.4	126.6 ± 18.5	0.741
DBP (mm Hg)	71 ± 11.5	71.5 ± 12.6	70.8 ± 11	0.739
**HTN (1/2/3 degree), *n* (%)**	**10/21/45** **7.9/16.6/35.7%**	**2/6/23** **5.1/15.3/58.9%**	**8/15/22** **9.1/17.2/25.2%**	**0.007**
Dyslipidemia, *n* (%)	92 (73%)	30 (79.4%)	61 (70%)	0.16
Smoking, *n* (%)	27 (21.4%)	9 (23%)	19 (21.8%)	0.541
Diabetes mellitus, *n* (%)	15 (11.9%)	5 (12.8%)	10 (11.4%)	0.379
**NYHA Class (II/III/IV), *n* (%)**	**81/16/1** **64.2/12.6/0.8%**	**31/6/0** **79.5/15.3/0%**	**50/10/1** **57.4/11.5/0.8%**	**0.008**
Angina (Class 1/2/3), *n* (%)	23/11/1 18.2/8.7/0.8%	10/2/0 25.6/5.1/0%	13/9/1 15/10.3/1.1%	0.395
Syncope, *n* (%)	16 (12.7%)	7 (17.9%)	9 (10.3%)	0.193
**HF worsening, *n* (%)**	**23 (18.2%)**	**15 (38.5%)**	**8 (9.2%)**	**0.001**
BNP baseline value, pg/ml median, (IQR)	170 (93–352)	252 (145–377)	139 (80–276)	0.131
**BNP Worsening, *n* (%)**	**25 (19.8%)**	**14 (35.9%)**	**11 (12.6%)**	**0.004**
**ECG characteristics**				
**PD (ms)**	**42 ± 16.4**	**57 ± 17.4**	**35.1 ± 10.1**	**<0.001**
**Pdur MAX (ms)**	**107.3 ± 16**	**118.4 ± 18.7**	**102.3 ± 11.7**	**<0.001**
**Pamp (mV)**	**0.24 ± 0.11**	**0.19 ± 0.05**	**0.26 ± 0.13**	**<0.001**
**Echocardiographic parameters**				
**LV parameters**				
MWT (mm)	20.9 ± 5.1	20 ± 4	21.3 ± 5.6	0.186
LV mass index (g/m^2^)	170.6 ± 63.2	182.8 ± 62.2	165.1 ± 63.3	0.137
LV EF (%)	67.5 ± 6.8	67.1 ± 6	67.7 ± 7.2	0.671
E/average e’	18.4 ± 8.2	19.6 ± 8.7	17.8 ± 8	0.258
LV GLS (%)	−14 ± 3.5	−13.7 ± 3.8	−14.2 ± 3.3	0.513
LV EDVi (ml/m^2^)	42.8 ± 12.3	44.2 ± 16.7	42.2 ± 9.8	0.486
LV ESVi (ml/m^2^)	13.9 ± 5.4	14.6 ± 6.5	13.6 ± 4.7	0.293
Diastolic dysfunction (Degree 1/2/3) *n* (%)	40/70/16 31.7/55.5/12.7%	9/22/8 23/56.4/20.5%	31/48/8 35.6/55.17/9.1%	0.281
**LA parameters**				
**LADi (mm/m^2^)**	**24.1 ± 3.5**	**25.3 ± 3.1**	**23.6 ± 3.5**	**0.009**
**LAVi (ml/m^2^)**	**62.2 ± 25.6**	**77.6 ± 31.9**	**55.2 ± 18.5**	**<0.001**
**LA strain (%)**	**16.6 ± 7.2**	**12.6 ± 6**	**18.4 ± 6.9**	**<0.001**
**LA SSR (s^–1^)**	**0.84 ± 0.43**	**0.65 ± 0.47**	**0.92 ± 0.37**	**<0.001**
LAESr (s**^–^**^1^)	−0.67 ± 0.33	−0.58 ± 0.29	−0.71 ± 0.34	0.053
**LA ASr (s^–1^)**	**-0.96 ± 0.52**	**-0.7 ± 0.33**	**-1.07 ± 0.55**	**<0.001**
**RV parameters**				
Free wall thickness (mm)	6.2 ± 1.7	6.2 ± 1.8	6.2 ± 1.6	0.906
TAPSE (mm)	23.3 ± 3.6	22.9 ± 3.9	23.4 ± 3.5	0.437
RV longitudinal strain (%)	−20.1 ± 4.9	−19.1 ± 5	−20.4 ± 4.8	0.211
FAC (%)	51 ± 8.1	51.2 ± 7.1	50.9 ± 8.4	0.865
**RA parameters**				
RA mediolateral diameter (mm)	36.4 ± 6.1	37.1 ± 7.8	36 ± 5.1	0.426
sPAP (mm Hg)	36.7 ± 11.44	38.29 ± 9.1	36 ± 12.2	0.339
**Resting LVOT gradient (mm Hg)**	**45 ± 42.5**	**58.6 ± 45.9**	**38.8 ± 39.6**	**0.013**
**Maximal LVOT gradient (mm Hg)**	**57.2 ± 45.5**	**72.9 ± 44.2**	**50.3 ± 44.6**	**0.01**
**MR severity (Degree 1/2/3/4, *n* %)**	**54/40/28/1** **42.8/31.7/22.2/0.8%**	**9/14/15/0** **23/35/38.5/0%**	**45/26/13/1** **51.7/29.9/14.9/1.1%**	**0.023**

*BMI, body mass index; SBP, systolic blood pressure; DBP, diastolic blood pressure; HTN, systolic arterial hypertension; NYHA, New York Heart Association; HF, heart failure; BNP, brain natriuretic peptide; IQR, interquartile range; PD, P wave dispersion; Pdur max, maximal duration of the P wave; Pamp, sum of maximal amplitude of P wave in lead V1, DII; LV, left ventricle, MWT, maximal wall thickness; EF, ejection fraction; GLS, global longitudinal strain; EDVi, indexed end diastolic volume; ESVi, indexed end sistolic volume; LADi – indexed left atrial anteroposterior diameter; LAVi – indexed left atrial maximal volume; LA, left atrium; LA SSR, left atrium systolic strain rate; LA ESr, left atrium early diastolic strain rate; LA ASr, left atrium late diastolic strain rate during atrial contraction; RV, right ventricle; TAPSE, tricuspid annular plane systolic excursion; FAC, fractional area change; sPAP, systolic pulmonary arterial pressure; LVOT, left ventricular outflow tract obstruction; and MR, mitral regurgitation. Bold values reflect that the difference between groups is statistically significant (p < 0.05).*

**FIGURE 1:**
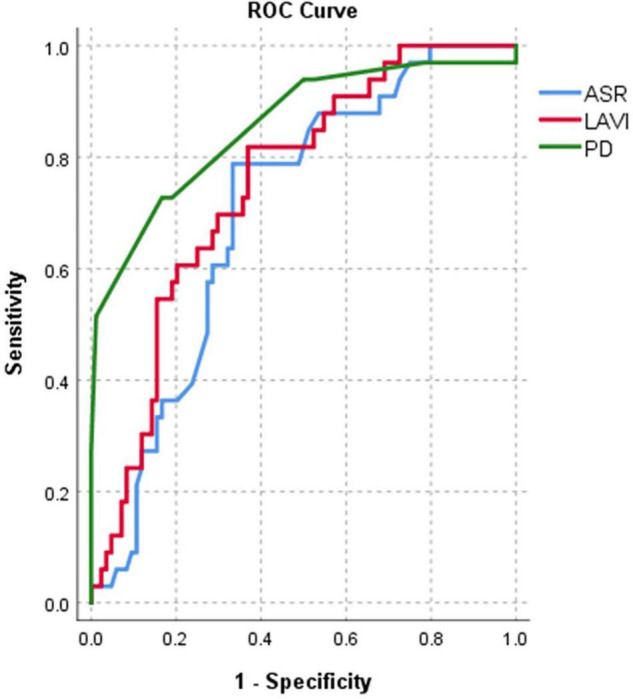
Area under curve (AUC)-based *C*-statistics for new-onset AF: for Pd (AUC = 0.86), LAVi (AUC = 0.76), and ASr (AUC = 0.7) in the whole population.

**FIGURE 2:**
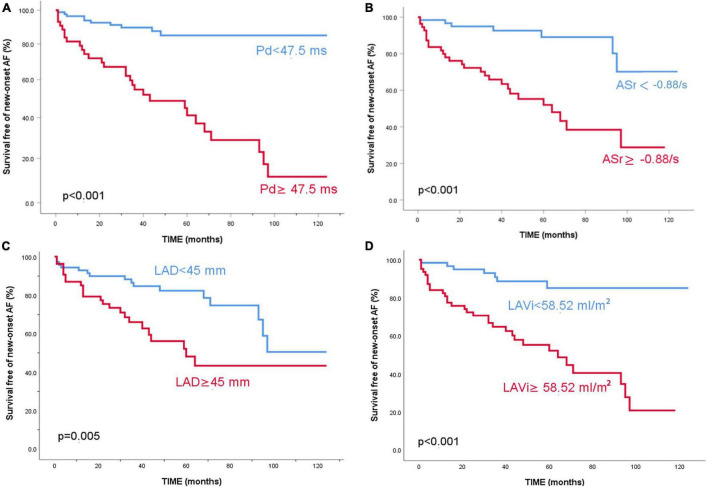
Kaplan–Meier survival curves free of new-onset atrial fibrillation for the entire study population. Patients are stratified according to P wave dispersion **(A)** 5-year survival free of events of 85% vs 41.2%, *p* < 0.001, ASr **(B)** 5-year survival free of events of 89.1% vs 52%, *p* < 0.001, LAD **(C)** 5-year survival free of events of 82.4% vs 48.1%, *p* = 0.005 and LAVi **(D)** 5-year survival free of events of 85.2% vs 52.2%, *p* < 0.001.

**FIGURE 3:**
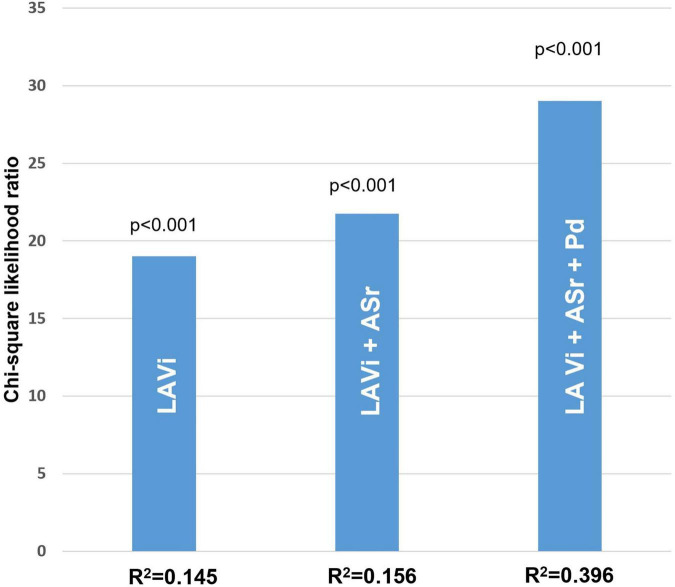
Likelihood ratio test in the whole HCM population. Significant incremental predictive value for new-onset AF when adding ASr, further improving when adding both ASr and Pd (model accuracy of 85%) compared to a standard model taking into account only LA indexed volume (model accuracy of 71%).

After Cox regression analysis, age, HTN severity, Pd, Pdur max, LADi, LAVi, LA strain, resting LVOT gradient, and MR severity were found as univariate predictors for AF occurrence in the whole HCM population (Table 2). To comparatively assess the accuracy of different parameters in identifying AF, ROC curves were constructed and the AUC values were calculated (Table 3 and Figure 1). Kaplan–Meier survival curves free from new AF episodes in the whole population for Pd, ASr, LAD, and LAVi are presented in Figure 2. The multivariable analysis included age, Pd, LAVi, ASr, resting LVOT gradient, mitral regurgitation degree (categorical), and HT degree (categorical). Only Pd (HR = 1.044, CI = 1.029–1.058, and *p* = 0.001) and ASr (HR = 4.244, CI = 1.847–9.751, and *p* = 0.038) emerged as independent predictors for AF in the whole population. Excluding the eight patients with a previous history of paroxysmal AF before enrollment did not change the results of univariable or multivariable analyses. Likelihood ratio test in this population showed a significant incremental predictive value for new-onset AF to a standard model containing LAVi > 58.5 ml/m^2^ when adding ASr, further improving when taking into account LAVi, ASr, and Pd (Figure 3).

**TABLE 2:** Independent predictors for incident AF in the whole HCM population.

Univariate Cox regression analysis				Multivariate Cox regression analysis
	
	HR	95% CI	*p*	*p*-value
Age	**1.032**	**1.010–1.055**	**0.003**	
HTN (1/2/3)	**1.585**	**1.233–2.054**	**<0.001**	
*NYHA class*			0.110	
PD*	**1.044**	**1.029–1.058**	**<0.001**	**0.001**
Pdur MAX*	**1.037**	**1.021–1.053**	**<0.001**	
Pamp**	**10^–4^**	**10^–4^–0.025**	**<0.001**	
LADi	**1.122**	**1.032–1.220**	**0.011**	
LAVi	**1.024**	**1.013–1.036**	**<0.001**	0.287
LA strain	**0.897**	**0.853–0.944**	**<0.001**	
LA ASr (s^–1^)	**4.244**	**1.847–9.751**	**<0.001**	**0.038**
Resting LVOT gradient	**1.009**	**1.001–1.016**	**0.022**	
*Maximal LVOT gradient*	*1.007*	*1–1.014*	**0.050**	
MR severity (1/2/3/4 degree)	**1.604**	**1.131–2.277**	**0.008**	

HTN, systemic hypertension; Pd, P wave dispersion; Pdur max, maximal duration of P wave; Pamp, sum of maximal amplitudes in lead DII, V1, LADi, indexed left atrial anteroposterior diameter; LAVi, indexed left atrial maximal volume; LA, left atrium; LA ASr, left atrium late diastolic strain rate during atrial contraction; LVOT, left ventricle outflow tract; and MR, mitral regurgitation. *HR for each increase in duration with 1 ms. **HR for each increase in amplitude with 1 mV. Bold values reflect that the difference between groups is statistically significant (p < 0.05).

**TABLE 3:** Electrocardiographic and echocardiographic predictors of new-onset AF with the prespecified cut-off values in the whole HCM group and in HCM patients with LAD < 45 mm.

Entire study population (*n* = 126)	AUC	*p*-value	Cut-off	Sensitivity, %	Specificity, %	NPV, %	PPV, %
Pd	0.86	<0.001	≥47.5 ms	74.4%	83.9%	88%	67.5%
LAVi	0.76	<0.001	≥58.5 ml/m^2^	81%	64.4%		
LA ASr	0.7	<0.001	≥−0.88 s^–1^	79.4%	66.7%	76.4%	51.66%
Patients with LAD < 45 mm (*n* = 72)							
Pd	0.89	<0.001	≥47.5 ms	87.5%	83.9%	95.7%	61.8%

*Pd, P wave dispersion; LAVi, indexed left atrial maximal volume; LA ASr, left atrium late diastolic strain rate during atrial contraction; NPV, negative predictive value; and PPV, positive predictive value. AUC, Area under curve.*

### Atrial Fibrillation Predictors in Patients With Left Atrium Diameter < 45 mm

Seventy-two patients had a LAD < 45 mm, considered by current guidelines as the cut-off for patients having an intermediate/lower risk for AF (3). However, the AF prevalence in this population was 22.2% (16/72 patients), comprising 41% of AF events in the total HCM population, still significantly higher than in the general population and surprisingly high for a population of HCM patients considered at low risk, in concordance with the results of other studies (4). Demographic, ECG, and echocardiographic characteristics of these patients are listed in Table 4.

**TABLE 4:** Demographic, clinical, ECG, and echocardiographic characteristics in patients with LAD < 45 mm, with and without AF.

	HCM patients with LAD < 45 mm and AF (*N* = 16)	HCM patients with LAD < 45 mm without AF (*N* = 56)	*p*
**Age (years)**	**62.4 ± 8.8**	**51.7 ± 18.3**	**0.024**
Men, *n* (%)	3 (18.7%)	23 (41%)	0.073
BMI (kg/m^2^)	27.6 ± 2.8	26.4 ± 5	0.216
SBP (mm Hg)	136 ± 29	128 ± 18	0.328
DBP (mm Hg)	25.5 ± 10.3	20.6 ± 9.9	0.736
**HTN (1/2/3 degree), *n* (%)**	**0/3/9** **0/18.7/56.2%**	**3/8/17** **5.3/14.2/30.3%**	**0.041**
Dyslipidemia, *n* (%)	12 (75%)	40 (71.4%)	0.431
Smoking, *n* (%)	3 (18.7%)	10 (17.8%)	0.622
Diabetes mellitus, *n* (%)	3 (18.7%)	4 (7%)	0.076
NYHA Class (II/III/IV), *n* (%)	13/3/0 81.2/18.7/0%	31/6/1 55.3/10.7/1.8%	0.109
Angina (Class 1/2), *n* (%)	5/2 31.2/12.5%	8/5 14.3/8.9%	0.395
Syncope, *n* (%)	4 (25%)	13 (23.2%)	0.265
**HF worsening, *n* (%)**	**5 (31.2%)**	**5 (8.9%)**	**0.044**
**BNP baseline value, pg/ml** **median, (IQR)**	**221** (137–350)	**139** (80–194)	**0.408**
BNP Worsening, *n* (%)	5 (31.2%)	9 (16%)	0.119
**ECG characteristics**			
**PD (ms)**	**58.2 ± 16.2**	**34.4 ± 10.5**	**<0.001**
**Pdur MAX (ms)**	**114.4 ± 11.1**	**101.5 ± 11.6**	**0.001**
**Pamp (mV)**	**0.19 ± 0.062**	**0.25 ± 0.10**	**0.028**
**Echocardiographic parameters**			
**LV parameters**			
MWT (mm)	18.8 ± 3.3	21.2 ± 5.2	0.075
LV mass index (g/m^2^)	169.9 ± 42	157.6 ± 52.5	0.380
LV EF (%)	68.9 ± 4.9	69 ± 7.4	0.990
E/average e’	21.7 ± 8.5	18.6 ± 9	0.226
LV GLS (%)	−14.8 ± 3.3	−14.3 ± 3.1	0.608
LV EDVi (ml/m^2^)	38.4 ± 7	40.4 ± 9.7	0.442
LV ESVi (ml/m^2^)	12 ± 2.5	12.4 ± 4.4	0.750
Diastolic dysfunction (Degree 1/2/3) *n* (%)	4/9/3 25/56.2/18.7%	23/26/6 41/46.4/10.7%	0.453
**LA Parameters**			
**LADi (mm/m^2^)**	**24 ± 2.4**	**22.5 ± 2.7**	**0.040**
**LAVi (ml/m^2^)**	**65.3 ± 12.8**	**49.6 ± 15.5**	**0.001**
**LA strain (%)**	**13.2 ± 7.2**	**19.3 ± 7.4**	**0.004**
**LA SSR**	**0.68 ± 0.52**	**1 ± 0.38**	**0.009**
LA ESr (s^–1^)	−0.58 ± 0.32	−0.71 ± 0.34	0.170
LA ASr (s^–1^)	−0.85 ± 0.35	−1.15 ± 0.57	0.059
**RV parameters**			
RV free wall thickness (mm)	6.2 ± 2.1	5.9 ± 1.5	0.604
TAPSE (mm)	22.5 ± 3.4	23 ± 3.6	0.588
RV longitudinal strain (%)	−20.3 ± 5.6	−20.7 ± 4.6	0.774
FAC (%)	52.5 ± 7	51.7 ± 8.8	0.737
**RA parameters**			
RA mediolateral diameter (mm)	35.3 ± 6.4	34.5 ± 4.3	0.623
sPAP (mm Hg)	34.5 ± 4.3	36 ± 13.7	0.989
**Resting LVOT gradient (mm Hg)**	**62.4 ± 44.4**	**36.8 ± 39.6**	**0.026**
**Maximal LVOT gradient (mm Hg)**	**78 ± 45.4**	**47.5 ± 46.9**	**0.024**
MR severity (Degree 1/2/3; %)	7/3/6 43.7/18.7/37.5%	31/16/6 55.3/28.5/10.7%	0.113

*BMI, body mass index; SBP, systolic blood pressure; DBP, diastolic blood pressure; HTN, systolic arterial hypertension;NYHA, New York Heart Association; HF, heart failure; BNP, brain natriuretic peptide; PD, P wave dispersion; Pdur max, maximal duration of the P wave; Pamp, sum of maximal amplitude of P wave in lead V1, DII; LV, left ventricle; MWT, maximal wall thickness; EF, ejection fraction; GLS, global longitudinal strain; EDVi, indexed end diastolic volume; ESVi, indexed end sistolic volume; LADi, indexed left atrial anteroposterior diameter; LAVi, indexed left atrial maximal volume; LA, left atrium; LA SSR, left atrium systolic strain rate; LA ESr, left atrium early diastolic strain rate; LA ASr, left atrium late diastolic strain rate during atrial contraction; RV, right ventricle; TAPSE, tricuspid annular plane systolic excursion; FAC, fractional area change; sPAP, systolic pulmonary arterial pressure; LVOT, left ventricular outflow tract obstruction; and MR, mitral regurgitation. Bold values reflect that the difference between groups is statistically significant (p < 0.05).*

**FIGURE 4:**
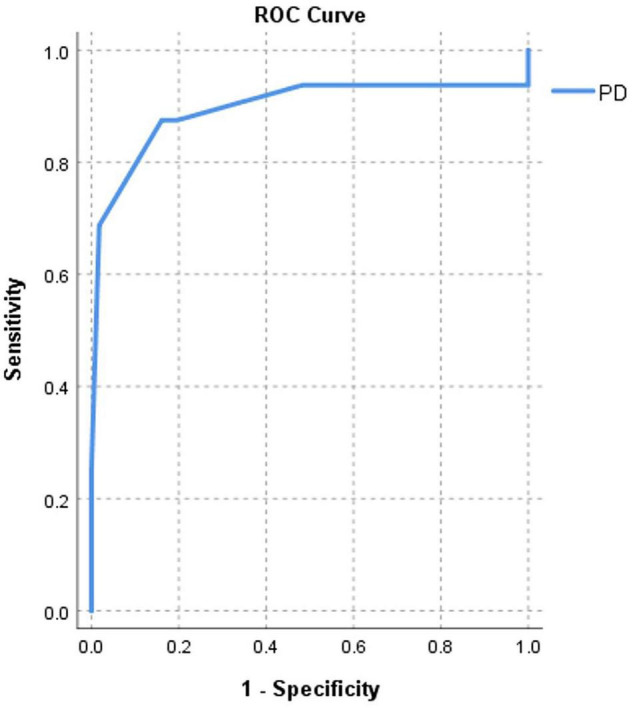
Area under curve (AUC)-based C-statistics for new-onset AF for Pd (AUC = 0.89) in HCM patients with LAD < 45 mm.

**FIGURE 5:**
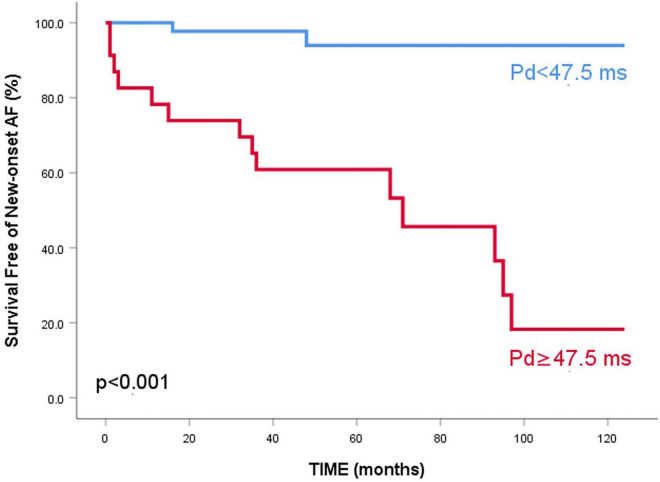
Kaplan–Meier survival curve free of new-onset atrial fibrillation for HCM patients with LAD < 45 mm, stratified according to *P* wave dispersion – 5-year survival free of events of 94% vs 60.9%, *p* < 0.001.

After Cox regression analysis, age, HTN severity, Pd, Pdur max, LADi, LAVi, LA strain, and resting LVOT gradient were univariate predictors for new-onset AF in this selected group. Pd emerged as the only AF predictor at multivariable analysis (HR = 1.105, 95% CI 1.059–1.154, and *p* = 0.002), independent of age, LAVi, LA strain, or resting LVOT gradient (Table 5). Exclusion of patients with a history of AF before enrollment did not change the univariable/multivariable analysis results. Pd had good accuracy in predicting AF, for a similar cut-off of 47.5 ms (Table 3 and Figure 4). Patients with a Pd < 47.5 ms had a better survival free of AF than patients with Pd ≥ 47.5 ms (Figure 5).

**TABLE 5:** Independent predictors for incident AF in HCM patients with LAD < 45 mm.

Univariate Cox regression analysis				Multivariate Cox regression analysis
	
	HR	95% CI	*p*	*p*-value
**Age**	**1.041**	**1.004–1.079**	**0.031**	
**HTN (1/2/3)**	**1.772**	**1.153–2.725**	**0.009**	
PD*	**1.105**	**1.059–1.154**	**<0.001**	**0.002**
Pdur MAX*	**1.061**	**1.012–1.112**	**0.013**	
*Pamp*			*0.054*	
**LADi**	**1.211**	**1.019–1.439**	**0.030**	
**LAVi**	**1.047**	**1.018–1.077**	**0.001**	
**LA strain**	**0.910**	**0.845–0.981**	**0.013**	
**Resting LVOT gradient**	**1.000**	**1.000–1.022**	**0.051**	
*Maximal LVOT gradient*			0.061	

HTN, systemic hypertension; Pd, P wave dispersion; Pdur max, maximal duration of P wave; Pamp, sum of maximal amplitudes in lead DII, V1, LADi, indexed left atrial anteroposterior diameter; LAVi, indexed left atrial maximal volume; LA, left atrium; LVOT, left ventricle outflow tract; and MR, mitral regurgitation. *HR for each increase in duration with 1 ms increase. Bold values reflect that the difference between groups is statistically significant (p < 0.05).

### The Relationship Between New-Onset Atrial Fibrillation and Heart Failure Worsening

Out of the 39 patients who experienced new-onset AF, 15 had worsening of HF symptoms. In the subgroup of patients with LAD < 45 mm, five out of the 16 patients with AF experienced worsening of their HF symptoms during follow-up and an increase in BNP levels. However, the correlation (Phi and Cramer’s V) between new-onset AF and HF worsening was weak both in the whole HCM study group (Phi = 0.35, *p* < 0.001) and in HCM patients with LAD < 45 mm (Phi = 0.27, *p* = 0.023).

## Discussion

This is the first study that simultaneously assessed the relationship between electrical (by ECG), structural and functional LA remodeling (by echocardiography) and AF occurrence in patients with HCM. The most important findings are: (1) LA size (LA diameter and volume), function (LA strain, LA booster pump function), and electrical parameters (Pd, Pdur, Pamp) are all related to AF occurrence. (2) LA booster pump function (ASr) and LA electrical dispersion (Pd) and not LA size (LA diameter or LA volume) emerged as independent predictors for incident AF. (3) In patients with LAD < 45 mm, the only independent predictor for AF was Pd, with good accuracy. Similar to other studies (4), the prevalence of AF in patients with LAD < 45 mm was significantly higher than in the general population.

Left atrial dilation is common in patients with HCM due to multiple underlying factors (24), such as changes in LV filling pressures (secondary to diastolic dysfunction), mitral regurgitation, and outflow tract obstruction (25–27). Pressure overload can negatively impact the LA, since the thin atrial wall is susceptible to increased wall tension, leading to atrial stretching and remodeling and, in turn, to atrial dilation (26, 27).

Current recommendations for arrhythmia screening with 48-h ECG Holter monitoring every 6 months in patients with LAD > 45 mm lack sensitivity (3). This is not surprising since the LA is a tridimensional structure, with dilation occurring in a non-uniform fashion. Slight variations in LAD can translate into significant volume changes, especially when significant LA dilation is present (4, 28). The current study confirms the superiority of LAVi over LADi in predicting AF, concordant with previous findings (1, 4, 29).

Whether LA enlargement is the cause of AF or merely an effect, or both, is difficult to establish. Atrial dilation leads to atrial stretch, which in turn may lead to an increase in electrical dispersion, a decrease in conduction, and to atrial remodeling and fibrosis, all being a substrate for AF. On the other hand, AF episodes decrease the atrial refractory period and promote LA dysfunction and dilation, initiating a vicious circle (29, 30).

After multivariable analysis, LAVi did not emerge as an independent predictor for AF in our study, possibly because most of our patients had significant LA dilation (median LAVi of 58 ml/m^2^). Thus, functional and electrical changes of the LA may refine risk stratification of AF in a population with important LA structural abnormalities. Moreover, specific HCM mutations might increase the susceptibility to AF, similar to ventricular arrhythmias (31, 32). This can translate into changes in LA electrical activity in the absence of significant LA structural remodeling.

### Left Atrial Dysfunction and Atrial Fibrillation

Abnormalities in LA function are common in patients with HCM, regardless of the presence of AF. Early stages of the disease are characterized by impairment of reservoir and conduit function, with preserved or even increased booster pump function as an adaptive initial response to LV diastolic dysfunction to maintain LV filling pressures (33, 34). A global myopathic process might also explain the impairment in LA function. Although the evidence for an intrinsic atrial myopathy is not strong, some work, including ours, showed a close relationship between LA function and LV remodeling, which might support this hypothesis (17, 35).

While Maron et al. demonstrated that LA total emptying fraction (with a cut-off value of less than 38%) is helpful in predicting AF in HCM patients (5), Losi et al. used LA global fractional shortening to identify patients at risk (29), and Debonnaire et al. proved that LA strain (cut-off of ≤23.4%) is a predictor for AF (4). However, there is currently no available data regarding the relationship between 2D strain parameters that evaluate each LA phasic function and the risk of AF in HCM. In our study, all three LA functions (reservoir, conduit, and booster pump) were significantly reduced in patients with AF, with ASr (reflecting contractile function) being the only LA functional parameter that independently predicted AF in our cohort of HCM patients.

### Atrial Electrical Dispersion and Atrial Fibrillation

Left atrial remodeling, atrial myopathy, and intrinsic electrophysiological abnormalities of the atria found in HCM patients are all factors leading to inhomogeneous propagation of atrial impulse, which predisposes to reentry. Pd is the expression of increased intra and interatrial anisotropy on surface ECG, which is a substrate for atrial arrhythmias and AF, thus an increase in Pd can be used as a reliable risk factor for AF occurrence (13, 36, 37).

Various studies have shown a direct link between an increased Pd and the risk for AF in different cardiovascular diseases (38–40). There are only two small retrospective studies about the role of Pd in patients with HCM, showing that a value of >46 ms was independently correlated with the risk for paroxysmal AF (8, 9). Our study is the first prospective study to prove a direct link between atrial dispersion and AF occurrence in HCM patients.

While other factors such as diabetes, obesity and renal failure can influence Pd (41, 42), there were no differences in the prevalence of these conditions between the AF and non-AF patients in our study. Age and hypertension can increase Pd, due to increased diastolic dysfunction and atrial fibrosis, respectively (40, 42). Nevertheless, Pd emerged as a strong independent predictor for AF in our study both in the general HCM population and in patients with LAD < 45 mm, with very weak correlations between Pd and age or hypertension. Adding both ASr and Pd to a model containing only LAD > 45 mm for predicting AF led to a significantly higher predictive value for new-onset AF in patients with HCM.

Moreover, Pd was the only LA remodeling parameter independently correlated with AF in HCM patients with a LAD < 45 mm and correlated poorly with structural and functional LA parameters (LAVi, LA strain, and ASr). A possible explanation might be that the initial changes in electrical activation and propagation of the action potential are more important in AF genesis than the changes in LA size or function (7, 40). The increase in P wave duration is often more related to the prolongation of interatrial conduction time than the actual increase in LA size (43). The fact that Pd is associated with the electrical activity of both atria, and the right atrium also contributes to AF, might be another explanation.

### Clinical Implications

Given that all studies showed a direct link between AF and worse outcomes in HCM patients, it is essential to identify patients at high arrhythmic risk (4, 10, 35). Moreover, AF duration is irrelevant for the thromboembolic risk, patients with a single AF episode having a similar risk compared to those with recurrent/persistent AF, oral anticoagulation being the only way to decrease this risk (35, 44).

While patients with LA diameter > 45 mm are clearly at risk of developing AF, LAVi, ASr, and Pd outperform LA diameter in accuracy, with higher sensitivity, specificity, PPV and NPV in identifying HCM patients at risk for AF. Moreover, only ASr and Pd emerged as independent risk factors associated with AF, suggesting these parameters may be preferred instead of LAD.

Several studies, including our own, proved that 20–50% of patients with LAD < 45 mm had at least one episode of AF (4). Therefore, identifying other AF determinants, especially in the so-called “low risk” group (LAD < 45 mm) can add incremental value over LAD, with clinical and prognostic implications. Pd can be determined by performing a simple ECG, so it can be easily implemented in clinical practice. Automatic measurement of Pd from electronically stored ECG is a fast and accurate alternative method (13, 14). Measuring LA strain by echocardiography is now widely available, recommended for both LA function and LV diastolic function assessment and can provide incremental information about the risk of AF in this setting (45, 46).

### Study Limitations

This study was performed in a single, tertiary center, so these results may not be directly translated to a general HCM population. The sample size was relatively small, which did not allow subgroup analysis on risk factors between patients with paroxysmal AF and those who developed persistent AF, but HCM is a relatively rare disease. The true prevalence of AF in our study group cannot be accurately determined since for most patients the diagnosis was made on surface ECG/Holter ECG recordings (47). Echocardiographic and ECG measurements were performed only at study enrollment, so we cannot account for the possible change in risk profile during study duration. We cannot establish whether LA electrical changes precede functional/structural changes, even if Pd emerged as the only independent predictor for AF in patients with LAD < 45 mm. LA deformation was assessed only in the apical four-chamber view, but this was done as recommended by current guidelines and care was taken to avoid LA foreshortening (23). Pd measurement was done manually but digital tools were used for better accuracy, as previously reported (14).

## Conclusion

Left atrium size (diameter and volume), function (LA strain, ASr), and electrical activity (Pd, Pamp) are all related to the risk of developing AF in HCM patients. Only ASr (reflecting LA contractile function) and Pd (reflecting LA electrical remodeling) emerged as independent predictors for new-onset AF, while in the subgroup of HCM patients with LAD < 45 mm, only Pd was independently associated with the presence of AF. Our findings suggest that the assessment of LA function and electrical activity can provide improved stratification of the risk for AF in HCM patients, including those considered at lower risk based on currently recommended risk parameters.

## Data Availability Statement

The original contributions presented in the study are included in the article/Supplementary Material, further inquiries can be directed to the corresponding author.

## Ethics Statement

The studies involving human participants were reviewed and approved by Ethics committee of the Emergency Institute for Cardiovascular Diseases “Prof. Dr. CC Iliescu”, Bucharest, Romania. The patients/participants provided their written informed consent to participate in this study.

## Author Contributions

LM, MR, and BP contributed to conception and design of the study. LM, MR, DC, CCB, RE, and ACă organized the database. LM, MR, and CB performed the statistical analysis. LM and MR wrote the first draft of the manuscript. ACu, CB, RJ, CG, and BP wrote sections of the manuscript. All authors contributed to manuscript revision, read, and approved the submitted version.

## Conflict of Interest

BP has received research support and lecture honoraria from GE Healthcare and Hitachi-Aloka. The remaining authors declare that the research was conducted in the absence of any commercial or financial relationships that could be construed as a potential conflict of interest.

## Publisher’s Note

All claims expressed in this article are solely those of the authors and do not necessarily represent those of their affiliated organizations, or those of the publisher, the editors and the reviewers. Any product that may be evaluated in this article, or claim that may be made by its manufacturer, is not guaranteed or endorsed by the publisher.

## Supplementary Material

Supplementary Figure 1 LA strain analysis by 2D-speckle echocardiography using a six segment model from the four chamber apical view. **(A)** Longitudinal strain rate curves with peak systolic (SSr), early diastolic (ESr) and late diastolic (Asr) strain rates, corresponding to LA reservoir, conduit, and booster pump functions. **(B)** Peak longitudinal LA strain for the assessment of LA reservoir function.

## References

[1] Nistri S Olivotto I Betocchi S Losi MA Valsecchi G Pinamonti B Prognostic significance of left atrial size in patients with hypertrophic cardiomyopathy (from the Italian Registry for Hypertrophic Cardiomyopathy). *Am J Cardiol.* (2006) 98:960–5. 10.1016/j.amjcard.2006.05.013 16996883

[2] Philipson DJ Rader F Siegel RJ. Risk factors for atrial fibrillation in hypertrophic cardiomyopathy. *Eur Jour of Prev Cardiol.* (2021) 28:658–65. 10.1177/2047487319828474 30727760

[3] Elliott PM Anastasakis A Borger MA Borggrefe M Cecchi F Charron P 2014 ESC Guidelines on diagnosis and management of hypertrophic cardiomyopathy: the task force for the diagnosis and management of hypertrophic cardiomyopathy of the European society of cardiology. *Eur Heart J.* (2014) 35:2733–79. 10.1093/eurheartj/ehu284 25173338

[4] Debonnaire P Joyce E Hiemstra Y Mertens BJ Atsma DE Schalij MJ Left atrial size and function in hypertrophic cardiomyopathy patients and risk of new-onset atrial fibrillation. *Circ Arrhythm Electrophysiol.* (2017) 10:e004052. 10.1161/CIRCEP.116.004052 28183843

[5] Maron BJ Haas TS Maron MS Lesser JR Browning JA Chan RH Left atrial remodeling in hypertrophic cardiomyopathy and susceptibility markers for atrial fibrillation identified by cardiovascular magnetic resonance. *Am J Cardiol.* (2014) 113:1394–400. 10.1016/j.amjcard.2013.12.045 24589281

[6] Tuluce K Tuluce YS Eren NK Kocabas U Akcay FA Gunduz R Predictors of future atrial fibrillation development in patients with hypertrophic cardiomyopathy: a prospective follow-up study. *Echocardiography.* (2016) 33:379–85. 10.1111/echo.13093 26493159

[7] Kose S Aytemir K Sade E Can I Ozer N Amasyali B Detection of patients with hypertrophic cardiomyopathy at risk for paroxysmal atrial fibrillation during sinus rhythm by P-wave dispersion. *Clin Cardiol.* (2003) 26:431–4. 10.1002/clc.4960260910 14524601 PMC6654222

[8] Ozdemir O Soylu M Demir AD Topaloglu S Alyan O Turhan H P-wave durations as a predictor for atrial fibrillation development in patients with hypertrophic cardiomyopathy. *Int J Cardiol.* (2004) 94:163–6. 10.1016/j.ijcard.2003.01.001 15093974

[9] Tjahjadi C Hiemstra YL van der Bijl P Pio SM Bootsma M Ajmone Marsan N Assessment of left atrial electro-mechanical delay to predict atrial fibrillation in hypertrophic cardiomyopathy. *Eur Heart J Cardiovasc Imaging.* (2021) 22:589–96. 10.1093/ehjci/jeaa174 32588037

[10] Olivotto I Cecchi F Casey SA Dolara A Traverse JH Maron BJ. Impact of atrial fibrillation on the clinical course of hypertrophic cardiomyopathy. *Circulation.* (2001) 104:2517–24. 11714644 10.1161/hc4601.097997

[11] Wilke I Witzel K Münch J Pecha S Blankenberg S Reichenspurner H High incidence of de novo and subclinical atrial fibrillation in patients with hypertrophic cardiomyopathy and cardiac rhythm management device. *J Cardiovasc Electrophysiol.* (2016) 27:779–84. 10.1111/jce.12982 27060297

[12] Williams B Mancia G Spiering W Rosei AE Azizi M Burnier M 2018 ESC/ESH Guidelines for the management of arterial hypertension. *Eur Heart J.* (2018) 39:3021–104. 30165516 10.1093/eurheartj/ehy339

[13] Gialafos JE Dilaveris PE Gialafos EJ Andrikopoulos GK Richter DJ Triposkiadis F P-wave dispersion: a valuable electrocardiographic marker for the prediction of paroxysmal lone atrial fibrillation. *Ann Noninvas Electrocardiol.* (1999) 4:39–45.

[14] Dilaveris P Batchvarov V Gialafos J Malik M. Comparison of different methods for manual P wave duration measurement in 12-lead electrocardiograms. *Pacing Clin Electrophysiol.* (1999) 22:1532–8. 10.1111/j.1540-8159.1999.tb00358.x 10588156

[15] Hindricks G Tatjana P Dagres N Arbelo E Bax JJ Blomstrom-Lundqvist C 2020 ESC Guidelines for the diagnosis and management of atrial fibrillation developed in collaboration with the European Association for Cardio-Thoracic Surgery (EACTS). *Eur Heart J.* (2020) 42:373–498. 10.1093/eurheartj/ehaa612 32860505

[16] Evangelista A Flachskampf F Lancellotti P Badano L Aguilar R Monaghan M European Association of Echocardiography recommendations for standardization of performance, digital storage and reporting of echocardiographic studies. *Eur J Echocardiogr.* (2008) 9:438–48. 10.1093/ejechocard/jen174 18579482

[17] Rosca M Popescu BA Beladan CC Calin A Muraru D Popa EC Left atrial dysfunction as a correlate of heart failure symptoms in hypertrophic cardiomyopathy. *J Am Soc Echocardiogr.* (2010) 23:1090–8. 10.1016/j.echo.2010.07.016 20739145

[18] Lang RM Badano LP Mor-Avi V Afilalo J Armstrong A Ernande L Recommendations for cardiac chamber quantification by echocardiography in adults: an update from the American Society of Echocardiography and the European Association of Cardiovascular Imaging. *Eur Heart J Cardiovasc Imaging.* (2015) 16:233–70. 25712077 10.1093/ehjci/jev014

[19] Cardim N Galderisi M Edvardsen T Plein S Popescu BA D’Andrea A Role of multimodality cardiac imaging in the management of patients with hypertrophic cardiomyopathy: an expert consensus of the European Association of Cardiovascular Imaging Endorsed by the Saudi Heart Association. *Eur Heart J Cardiovasc Imaging.* (2015) 16:280. 10.1093/ehjci/jeu291 25650407

[20] Nagueh SF Smiseth OA Appleton CP Byrd BF Dokainish H Edvardsen T Recommendations for the evaluation of left ventricular diastolic function by echocardiography: an update from the American society of echocardiography and the European association of cardiovascular imaging. *Eur Heart J Cardiovasc Imaging.* (2016) 17:1321–60. 27422899 10.1093/ehjci/jew082

[21] Lancellotti P Moura L Pierard LA Agricola E Popescu BA Tribouilloy C European Association of Echocardiography. European Association of Echocardiography recommendations for the assessment of valvular regurgitation. Part 2: mitral and tricuspid regurgitation (native valve disease). *Eur J Echocardiogr.* (2010) 11:307–32. 10.1093/ejechocard/jeq031 20435783

[22] Serri K Reant P Lafitte M Berhouet M Le Bouffos V Roudaut R Global and regional myocardial function quantification by two-dimensional strain; application in hypertrophic cardiomyopathy. *J Am Coll Cardiol.* (2006) 47:1175–81. 10.1016/j.jacc.2005.10.061 16545649

[23] Badano LP Kolias TJ Muraru D Abraham TP Aurigemma G Edvardsen T Standardization of left atrial, right ventricular, and right atrial deformation imaging using two-dimensional speckle tracking echocardiography: a consensus document of the EACVI/ASE/Industry Task Force to standardize deformation imaging. *Eur Heart J Cardiovasc Imaging.* (2018) 19:591–600. 10.1093/ehjci/jey042 29596561

[24] Yang H Woo A Monakier D Jamorski M Fedwick K Wigle ED Enlarged left atrial volume in hypertrophic cardiomyopathy: a marker for disease severity. *J Am Soc Echocardiogr.* (2005) 18:1075–82. 10.1016/j.echo.2005.06.011 16198885

[25] Greenberg B Chatterjee K Parmley WW Werner JA Holly AN. The influence of left ventricular filling pressure on atrial contribution to cardiac output. *Am Heart J.* (1979) 98:742–51. 10.1016/0002-8703(79)90473-3 495426

[26] Pritchett AM Mahoney DW Jacobsen SJ Rodeheffer RJ Karon BL Redfield MM. Diastolic dysfunction and left atrial volume: a population based study. *J Am Coll Cardiol.* (2005) 45:87–92. 10.1016/j.jacc.2004.09.054 15629380

[27] Sachdev V Shizukuda Y Brenneman CL Birdsall CW Waclawiw MA Arai AE Left atrial volumetric remodeling is predictive of functional capacity in nonobstructive hypertrophic cardiomyopathy. *Am Heart J.* (2005) 149:730–6. 10.1016/j.ahj.2004.07.017 15990760

[28] Tani T Tanabe K Ono M Yamaguchi K Okada M Sumida T Left atrial volüme and the risk of paroxysmal atrial fibrillation in patients with hypertrophic cardiomyopathy. *J Am Soc Echocardiogr.* (2004) 17:644–8. 15163936 10.1016/j.echo.2004.02.010

[29] Losi MA Betocchi S Aversa M Lombardi R Miranda M D’Alessandro G Determinants of atrial fibrillation development in patients with hypertrophic cardiomyopathy. *Am J Cardiol.* (2004) 94:895–900. 10.1016/j.amjcard.2004.06.024 15464672

[30] Satoh T Zipes DP. Unequal atrial stretch in dogs increases dispersion of refractoriness conductive to developing atrial fibrillation. *J Cardiovasc Electrophysiol.* (1996) 7:833–42. 10.1111/j.1540-8167.1996.tb00596.x 8884512

[31] Gruver EJ Fatkin D Dodds GA Kisslo J Maron BJ Seidman JG Familial hypertrophic cardiomyopathy and atrial fibrillation caused by Arg663His beta-cardiac myosin heavy chain mutation. *Am J Cardiol*. (1999) 83:13H–8H. 10.1016/s0002-9149(99)00251-9 10750581

[32] Girolami F Iascone M Tomberli B Bardi S Benelli M Marseglia G Novel α-actinin 2 variant associated with familial hypertrophic cardiomyopathy and juvenile atrial arrhythmias: a massively parallel sequencing study. *Circ Cardiovasc Genet.* (2014) 7:741–50. 10.1161/CIRCGENETICS.113.000486 25173926

[33] Anwar AM Soliman OI Nemes A Geleijnse ML ten Cate FJ. An integrated approach to determine left atrial volume, mass and function in hypertrophic cardiomyopathy by two-dimensional echocardiography. *Int J Cardiovasc Imaging.* (2008) 24:45–52. 10.1007/s10554-007-9224-x 17541727 PMC2121119

[34] Anwar AM Soliman OI Geleijnse ML Michels M Vletter WB Nemes A Assessment of left atrial ejection force in hypertrophic cardiomyopathy using real-time three-dimensional echocardiography. *J Am Soc Echocardiogr.* (2007) 20:744–8. 10.1016/j.echo.2006.11.017 17543746

[35] Guttmann OP Rahman MS O’Mahony C Anastasakis A Elliot PM. Atrial fibrillation and thromboembolism in patients with hypertrophic cardiomyopathy: systematic review. *Heart.* (2014) 100:465–72. 10.1136/heartjnl-2013-304276 24014282

[36] Tanigawa M Fukatani M Konoe A Isomoto S Kadena M Hashiba K. Prolonged and fractionated right atrial electrograms during sinus rhythm in patients with paroxysmal atrial fibrillation and sick sinus node syndrome. *J Am Coll Cardiol.* (1991) 17:403–8. 10.1016/s0735-1097(10)80106-8 1991897

[37] Prajapat L Ariyarajah V Frisella ME Apiyasawat S Spodick DH. Association of P-wave duration, dispersion, and terminal force in relation to P-wave axis among outpatients. *Ann Noninvasive Electrocardiol.* (2007) 12:210–5. 10.1111/j.1542-474X.2007.00163.x 17617065 PMC6932059

[38] Dilaveris PE Gialafos EJ Sideris SK Theopistou AM Andrikopoulos GK Kyriakidis M Simple electrocardiographic markers for the prediction of paroxysmal idiopathic atrial fibrillation. *Am Heart J.* (1998) 135:733–8. 10.1016/s0002-8703(98)70030-4 9588401

[39] Ciaroni S Cuenoud L Bloch A. Clinical study to investigate the predictive parameters for the onset of atrial fibrillation in patients with essential hypertension. *Am Heart J.* (2000) 139:814–9. 10.1016/s0002-8703(00)90012-7 10783214

[40] Aytemir K Ozer N Atalar E Sade E Aksoyek S Ovunc K P wave dispersion on 12-lead electrocardiography in patients with paroxysmal atrial fibrillation. *Pacing Clin Electrophysiol.* (2000) 23:1109–12. 10.1111/j.1540-8159.2000.tb00910.x 10914366

[41] Ertem AG Erdogan M Keles EM Durmaz T Bozkurt E. P-wave dispersion and left ventricular diastolic dysfunction in hypertension. *Anatol J Cardiol.* (2015) 15:78–9. 10.5152/akd.2014.5748 25550255 PMC5336910

[42] Demir K Avci A Kaya Z Marakoglu K Ceylan E Yilmaz A Assessment of atrial electromechanical delay and P-wave dispersion in patients with type 2 diabetes mellitus. *J Cardiol.* (2016) 67:378–83. 26164686 10.1016/j.jjcc.2015.06.003

[43] Hopkins CB Barret O. Electrocardiographic diagnosis of left atrial enlargement. Role of the P terminal force in lead V1. *J Electrocardiol.* (1989) 22:359–63. 10.1016/0022-0736(89)90012-5 2529337

[44] Maron BJ Olivotto I Bellone P Conte MR Cecchi F Flygenring BP Clinical profile of stroke in 900 patients with hypertrophic cardiomyopathy. *J Am Coll Cardiol.* (2002) 39:301–7. 10.1016/s0735-1097(01)01727-2 11788223

[45] Thomas L Marwick TH Popescu BA Donal E Badano LP. Left atrial structure and function, and left ventricular diastolic dysfunction: JACC state-of-the-art review. *J Am Coll Cardiol.* (2019) 73:1961–77. 10.1016/j.jacc.2019.01.059 31000000

[46] Smiseth OA Morris DA Cardim N Cikes M Delgado V Donal E Multimodality imaging in patients with heart failure and preserved ejection fraction: an expert consensus document of the European Association of Cardiovascular Imaging. *Eur Heart J Cardiovasc Imaging.* (2022) 23:e34–61. 10.1093/ehjci/jeab154 34729586

[47] Jorfida M Antolini M Cerrato E Caprioli MG Castagno D Garrone P Cryptogenic ischemic stroke and prevalence of asymptomatic atrial fibrillation: a prospective study. *J Cardiovasc Med.* (2016) 17:863–9. 10.2459/JCM.0000000000000181 25379716

